# Circulating lncRNA- and miRNA-Associated ceRNA Network as a Potential Prognostic Biomarker for Non-Hodgkin Lymphoma: A Bioinformatics Analysis and a Pilot Study

**DOI:** 10.3390/biomedicines10061322

**Published:** 2022-06-04

**Authors:** Mara Fernandes, Herlander Marques, Ana Luísa Teixeira, Rui Medeiros

**Affiliations:** 1 Molecular Oncology and Viral Pathology Group, Research Center of IPO Porto (CI-IPOP)/RISE@CI-IPOP (Health Research Network), Portuguese Oncology Institute of Porto (IPO Porto)/Porto Comprehensive Cancer Center (Porto.CCC), 4200-072 Porto, Portugal; mara.aires.fernandes@ipoporto.min-saude.pt (M.F.); ana.luisa.teixeira@ipoporto.min-saude.pt (A.L.T.); 2 Research Department of the Portuguese League against Cancer Regional Nucleus of the North (LPCC-NRN), 4200-177 Porto, Portugal; 3 Faculty of Medicine, University of Porto (FMUP), 4200-319 Porto, Portugal; 4 Life and Health Sciences Research Institute (ICVS), School of Medicine, Campus de Gualtar, University of Minho, 4710-057 Braga, Portugal; herlander.marques@hb.min-saude.pt; 5 ICVS/3B’s—PT Government Associate Laboratory, 4805-017 Braga/Guimarães, Portugal; 6 Department of Oncology, Hospital de Braga, 4710-069 Braga, Portugal; 7 CINTESIS, Center for Health Technology and Services Research, Faculty of Medicine, University of Porto, 4200-450 Porto, Portugal; 8 ICBAS–Instituto de Ciências Biomédicas Abel Salazar, Universidade do Porto, 4050-513 Porto, Portugal; 9 Biomedical Research Center (CEBIMED), Faculty of Health Sciences of Fernando Pessoa University (UFP), 4249-004 Porto, Portugal

**Keywords:** non-Hodgkin lymphoma, miRNA, lncRNA, ceRNA network, prognosis, biomarker

## Abstract

Non-Hodgkin lymphoma (NHL) is characterized by a great variability in patient outcomes, resulting in the critical need for identifying new molecular prognostic biomarkers. This study aimed to identify novel circulating prognostic biomarkers based on an miRNA/lncRNA-associated ceRNA network for NHL. Using bioinformatic analysis, we identified the miRNA-lncRNA pairs, and using RT-qPCR, we analyzed their plasma levels in a cohort of 113 NHL patients to assess their prognostic value. Bioinformatic analysis identified SNHG16 and SNHG6 as hsa-miR-20a-5p and hsa-miR-181a-5p sponges, respectively. Plasma levels of hsa-miR-20a-5p/SNHG16 and hsa-miR-181a-5p/SNG6 were significantly associated with more aggressive disease and IPI/FLIPI scores. Moreover, we found that patients with risk expression profiles of hsa-miR-20a-5p/SNHG16 and hsa-miR-181a-5p/SNHG6 presented a higher risk of positive bone marrow involvement. Moreover, hsa-miR-20a-5p/SNHG16 and hsa-miR-181a-5p/SNHG6 pairs’ plasma levels were associated with overall survival and progression-free survival of NHL patients, being independent prognostic factors in a multivariate Cox analysis. The prediction models incorporating the ceRNA network expression analysis improved the predictive capacity compared to the model, which only considered the clinicopathological variables. There are still few studies on using the ceRNA network as a potential prognostic biomarker, particularly in NHL, which may permit the implementation of a more personalized management of these patients.

## 1. Introduction

Non-Hodgkin lymphomas (NHLs) are a heterogenous group of lymphoproliferative malignancies in which the majority of cases arise from B cells during different stages of normal B-cell differentiation [1]. The latest GLOBOCAN data from 2020 indicate that NHL represents the most common hematological malignancy worldwide, corresponding to approximately 6% of cancer diagnoses and 3% of cancer deaths [2]. The most prevalent subtypes of NHL are the aggressive diffuse large B-cell lymphoma (DLBCL) and the indolent follicular lymphoma (FL), accounting for about 65% of all B-cell NHLs [3]. The combined anthracycline-containing chemotherapy (cyclophosphamide, doxorubicin, vincristine, prednisone–CHOP) with an anti-CD20 agent, Rituximab (R-CHOP), remains the standard therapy regime for NHL treatment [3]. Despite the improvement in patients’ outcomes, approximately 20–50% of patients are refractory ab initio or relapse at a later stage, which is alarmingly associated with a poor response to following chemotherapy lines, presenting only a 20–40% 2-year overall survival rate [4,5,6]. In clinical practice, the International Prognostic Index (IPI) is currently used to predict the outcome of NHL patients, classifying them into risk groups with different ranges of different overall-survival-based clinical factors [7,8]. Nevertheless, the very heterogenous nature of NHL, which is due to various genetic abnormalities and clinical features, results in highly variable treatment responses and ultimately unpredictable outcomes, even within the individual risk groups. Therefore, the unpredictability in patients’ outcome indicates there is a clinical need for additional biological biomarkers allowing for accurate prediction of prognoses and the monitoring of treatment response. Moreover, one of the established parameters that influences NHL patients’ prognoses is bone marrow involvement (BMI), which is correlated with a worse prognosis. Currently, BM aspiration and biopsy remain the gold standards for BM staging. However, not only are these procedures are considered invasive, raising the question of their routine use, especially in patients with a low probability of BMI, but they also do not necessarily reflect the overall condition of the BMI [9]. Therefore, given the relevance of assessing BMI in patients’ prognoses, there is a need to introduce new methodologies and improve the current methods. In this instance, it would be interesting to identify new molecular biomarkers to analyze in liquid biopsies for diagnosing BMI, allowing for the convenient and continuous collection and the ability to dynamically monitor patients to assess prognosis and response to treatment, guide treatment and detect early recurrence.

In recent years, an increasing number of non-coding RNAs (ncRNAs), including microRNAs (miRNAs) and long non-coding RNAs (lncRNAs), have emerged as attractive biomarker candidates with clinical potential [10]. Due to their intrinsic stability, they can be detected not only in tissue samples but also in biological fluids, such as blood, which opens a great opportunity to monitor the kinetics of the disease using a non-invasive approach [11].

MiRNAs are a class of small ncRNAs ~22 nucleotides in length that act as central post-transcriptional regulators of gene expression. MiRNAs bind to the 3′ untranslated region (UTR) of a target mRNA, resulting in their repression or degradation [12,13]. Despite the majority of studies being focused on solid tumors, the deregulation of miRNA expression has also been reported in lymphomas [14].

On the other hand, a new regulatory layer was added to the intricate regulation of gene expression involving lncRNAs, a >200 nt-long transcript [15]. In fact, the deregulation of lncRNAs is already associated with the development and progression of lymphoma [16]. LncRNAs perform an intricate regulatory function acting at different levels including epigenetic, transcriptional, post-transcriptional and translation regulation, as well as post-translational modification [17]. Recently, Salmena et al. proposed a new form of lncRNA-mediated regulation through a “competing endogenous RNA (ceRNA) network”, where lncRNAs function as endogenous molecular miRNA sponges, resulting in the release of mRNAs from the inhibitory action of miRNAs [18]. For example, lncRNA LINC01857 promotes cell proliferation and suppresses apoptosis of DLBCL cells by modulating the miR-141-3p/MAP4K4 axis [19]. Although most lncRNAs have not yet been functionally characterized, they have been showing potential for use as prognostic biomarkers, similarly to miRNAs [20,21].

The study of the interaction between miRNAs and lncRNAs is still in its infancy, especially in NHL, with the underlying molecular mechanisms remaining largely unclear. Moreover, additional studies are needed to further establish ncRNAs as potential new predictive and prognostic biomarkers for use in clinical practice in order to improve NHL patients’ management. Therefore, in the present study, we analyzed the potential of miRNAs and lncRNAs as prognostic biomarkers of NHL patients by investigating the plasma expression levels of has-miR-20a-5p ahashsa-miR-181a-5p and their respective lncRNA with which they form a ceRNA network. Hsa-miR-20a-5p is one of the components of the miR-17-92 cluster (comprising miR-17, miR-18a, miR-19a, miR-19b-1, miR-20a and miR-92-1), which was demonstrated to play a central role during the stages of B-cell development, and its deregulated expression was shown to have oncogenic potential [22,23,24]. In fact, B-cell-specific miR-17∼92 transgenic mice developed lymphomas with high penetrance which phenotypically resemble human lymphomas, including DLBCL, demonstrating the role of this cluster as a driver of B-cell lymphomagenesis [25]. Similarly, miR-181a-5p was also shown to be differentially expressed during the development stages of B cells; in particular, miR-181a-5p ectopic overexpression in common lymphoid progenitors results in increasing the total number of B cells, demonstrating its role in the fine-tuning of B-cell development [26,27,28].

## 2. Materials and Methods

### 2.1. Construction of CeRNA Regulatory Network and Functional Analysis

StarBase database (https://starbase.sysu.edu.cn/ (accessed on 30 January 2020)) was used to determine miRNA-lncRNA interactions by applying the following parameters: clade: mammal, genome: human, assembly: hg19, number of supporting experiments: ≥3, pan-cancer ≥ 1 [29].

The miRNA-targeted mRNAs, which were only validated by strong evidence methods, were retrieved using miRTarBase (https://bio.tools/mirtarbase (accessed on 3 June 2021)) [30]. The protein–protein interaction (PPI) networks were analyzed using the Search Tool for the Retrieval of Interacting Genes (STRING) database. Construction and visualization of the protein interaction network of the selected target genes were realized using the STRINGapp of the Cytoscape software (v3.8.2, Cytoscape Consortium, San Diego, CA, USA)). STRING enrichment analysis tool was used to retrieve the functional enrichment analysis of Gene Ontology (GO), Kyoto Encyclopedia of Genes and Genomes (KEGG) and Reactome pathways. The enrichment results were filtered, and redundant terms were removed according to the Jaccard index. Cytoscape visualization software (https://cytoscape.org/, (accessed on 10 September 2021)) was used to construct the final interaction networks [31].

### 2.2. Study Population

The study included 113 patients diagnosed with B-cell NHL (high-grade lymphomas versus low-grade lymphomas), of Caucasian ethnicity, older than 18 years and without known familial cancer history, as described in a previous study [32,33]. Patients were admitted and treated at a Portuguese hospital between January 2016 and June 2020. Patients’ clinical information is summarized in Table 1.

### 2.3. RNA Extractions and qPCR

Blood samples were obtained at baseline before starting therapy. Peripheral blood samples were centrifuged for preparation of platelet-free plasma (PFP) samples, as previously described [34]. Supernatant was aliquoted and stored at −80 °C until use. The GRS microRNA kit (Grisp^®^, GRiSP Research Solutions, Porto, Portugal) was used to isolate the miRNA portion according to laboratory procedures, while Plasma/Serum RNA Purification Kit (Norgen^®^, Biotek Corp. Thorold, ON, Canada)) was used for total RNA isolation, according to manufacturer’s instructions. Using NanoDrop Lite spectrophotometer (Thermo Scientific^®,^, Waltham, MA, USA), the RNA concentration and purity were determined. Taqman^®^ MicroRNA Reverse Transcription kit along with Taqman^®^ MicroRNA assay (Applied Biosystems^®^, Waltham, MA, USA) were used to carry out the cDNA synthesis for the miRNAs. For the analysis of lncRNA expression, the cDNA synthesis was performed using the High-Capacity cDNA Reverse Transcription Kit (Applied Biosystems^®^, Waltham, MA, USA in accordance with manufacturer’s instructions. Using qPCR, the miRNA and lncRNA expression levels were quantified using a StepOneTM qPCR Real-Time PCR machine, and the following reaction mix components: 1xTaqMan^®^ Gene Expression Master mix (Thermo Fisher Scientific^®^) and 1x probes TaqMan^®^ MicroRNA Assays (hsa-miR-20a-5p: 000580 and hsa-miR181a-5p: 000480) and TaqMan^®^ Noncoding RNA assays (SNHG16: Hs01598403_g1 and SNHG6: Hs00417251_m1) (Applied Biosystems^®^, Waltham, MA, USA). MiRNAs expression levels were normalized to hsa-miR-16 (000391), and lncRNA expression levels were normalized to GAPDH (Hs99999905_m1) endogenous control. Each sample had two technical replicates. The amplification conditions were: a holding stage 95 °C for 20s, followed by 45 cycles of 95 °C for 1s and 60 °C for 20 s. Data analysis was performed using StepOneTM Sofware v2.2 (Applied Biosystems^®^, Waltham, MA, USA), with the same baseline and threshold set for each plate, to generate threshold cycle (Ct) values for all the genes in each sample.

### 2.4. Statistical Analysis

Statistical analysis was performed using SPSS (version 26.0; IBM Company, Chicago, IL, USA) and GraphPad Prism software (version 7.0; San Diego, CA, USA). Student’s t-test or Mann–Whitney U test was used to evaluate statistical differences in the normalized expression (−ΔCt) of the miRNAs and lncRNAs among the different groups. Additionally, the 2^−ΔΔCt^ method (Livak method) was used to calculate the relative changes in gene expression between the different groups.

Receiver operating characteristic (ROC) analysis was performed to assess the prognostic accuracy, and the AUC was calculated. Overall survival (OS) and progression-free survival (PFS) curves were generated using Kaplan–Meier method and compared using the log-rank test. OS time was determined from the date of diagnosis to the date of mortality or the last follow-up. PFS time was determined to extend from the date of diagnosis to the date of disease progression, recurrence, mortality or last follow-up. Cox regression was used to analyze the prognostic value of the miRNAs/lncRNAs expression levels on the progression-free and overall survival. *p* < 0.05 was considered statistically significant.

## 3. Results

### 3.1. lncRNA-miRNA-mRNA Network Construction

**Figure 1:**
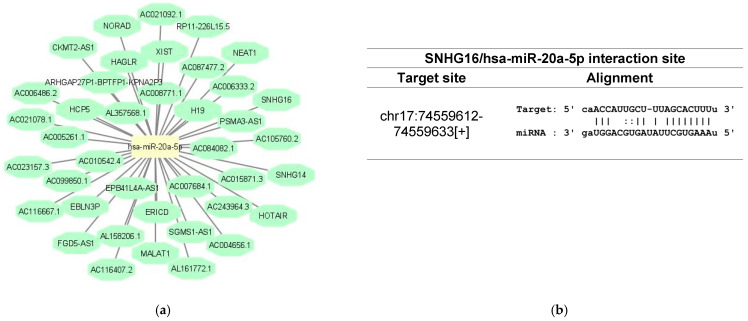

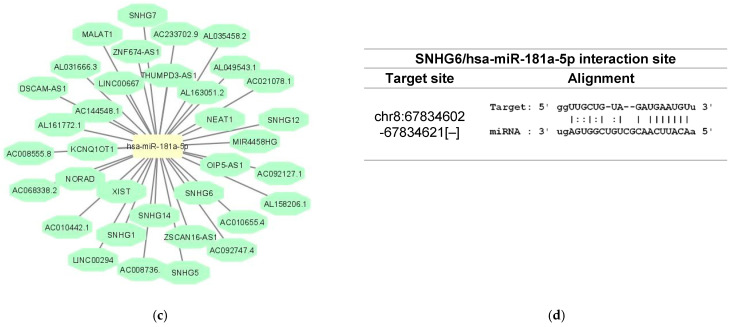
In silico analysis of the lncRNAs targeting hsa-miR-20a-5p and hsa-miR-181a-5p. (**a**) lncRNAs that target hsa-miR-20a-5p according to StarBase database analysis; (**b**) details about the binding site of hsa-miR-20a-5p on SNHG16, predicted by StarBase database; (**c**) lncRNAs that target hsa-miR-181a-5p according to StarBase database analysis; (**d**) details about the binding site of hsa-miR-181a-5p on SNHG6, predicted by StarBase database.

LncRNAs have the ability to sponge a variety of miRNAs to inhibit their regulatory effect on target mRNAs, creating a ceRNA network [18]. The StarBase database was used to identify the lncRNAs that target hsa-miR-20a-5p and hsa-miR-181a-5p (Figure 1). The analysis identified 40 lncRNAs targeting hsa-miR-20a-5p and 35 lncRNAs targeting hsa-miR-181a-5p. For the subsequent analysis, we filtered the results by gene type=processed transcript and gene name = SNHG to obtain the respective lncRNAs from the biotype small nucleolar RNA host gene (SNHG), an emergent class of lncRNAs that have been involved in the induction of proliferation, cell cycle progression, invasion and metastasis of cancer cells, making this class of transcripts a viable and attractive biomarker for cancer development and progression [33].

**Figure 2:**
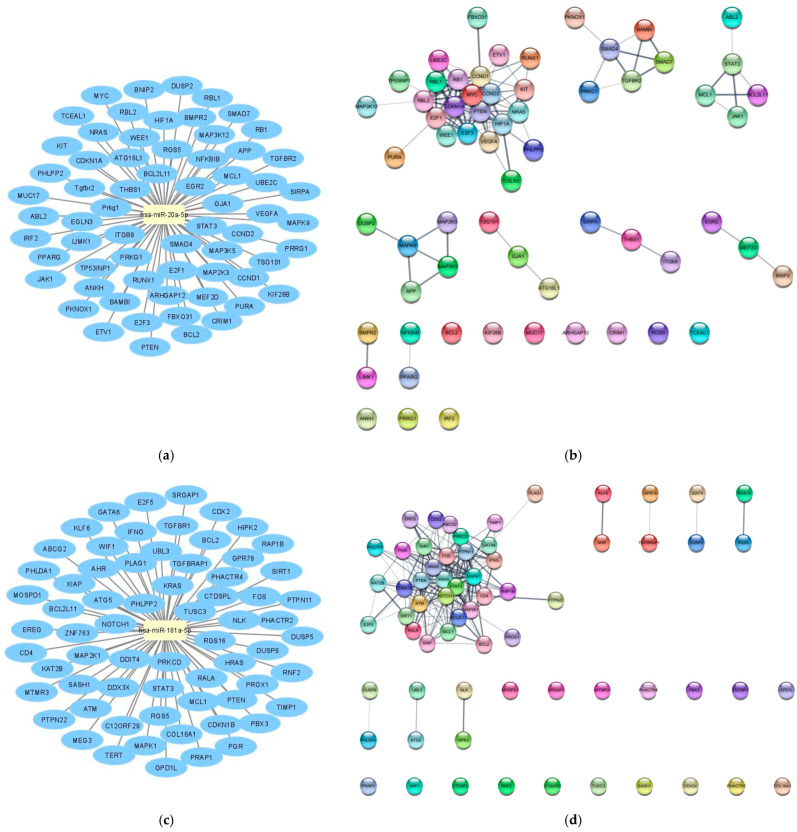
In silico analysis of the target mRNAs of hsa-miR-20a-5p and hsa-miR-181a-5p. (**a**) hsa-miR-20a-5p target mRNAs according to miRTarBase database analysis; (**b**) target mRNAs of hsa-miR-20a-5p organized by string interactions clusters; (**c**) hsa-miR-181a-5p target mRNAs according to miRTarBase database analysis; (**d**) target mRNAs of hsa-miR-181a-5p organized by string interactions clusters.

**Figure 3:**
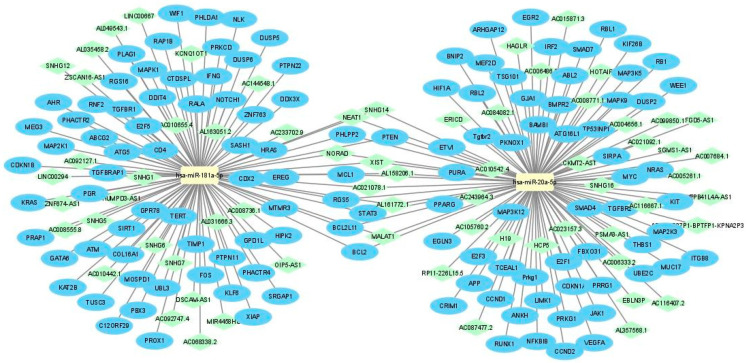
Data integration of the lncRNA–miRNA–mRNA networks related to hsa-miR-20a-5p and hsa-miR-181a-5p.

Subsequently, the mirTarBase database, the largest known online database of validated miRNA:mRNA interactions, was used to identify target mRNAs of hsa-miR-20a-5p and hsa-miR-181a-5p. According to miRTarBase analysis, we filtered the list of mRNAs by Homo Sapiens species and retrieved the interactions that were only validated with strong evidence methods (Western blot, qRT-PCR or luciferase assay), which are listed in Figure 2. In order to perform the functional annotation and enrichment analysis, we analyzed the validated targets with the STRINGapp Protein Query from Cytoscape software. The identified targets were filtered into a protein–protein interaction (PPI) network with 63 nodes and 270 edges for hsa-miR-20a-5p targets and 66 nodes and 256 edges for the hsa-miR-181a-5p targets, presenting a significant enrichment (*p* = 1 × 10^−16^ and *p* = 1 × 10^−16^, respectively). We also applied Markov clustering (MCL), which resulted in the clustering of the proteins according to their STRING interaction score (Figure 2b,d). Finally, we integrated the data from hsa-miR-20a-5p and hsa-miR-181a-5p identified targets and constructed the complete lncRNA-miRNA-mRNA network (Figure 3).

**Figure 4:**
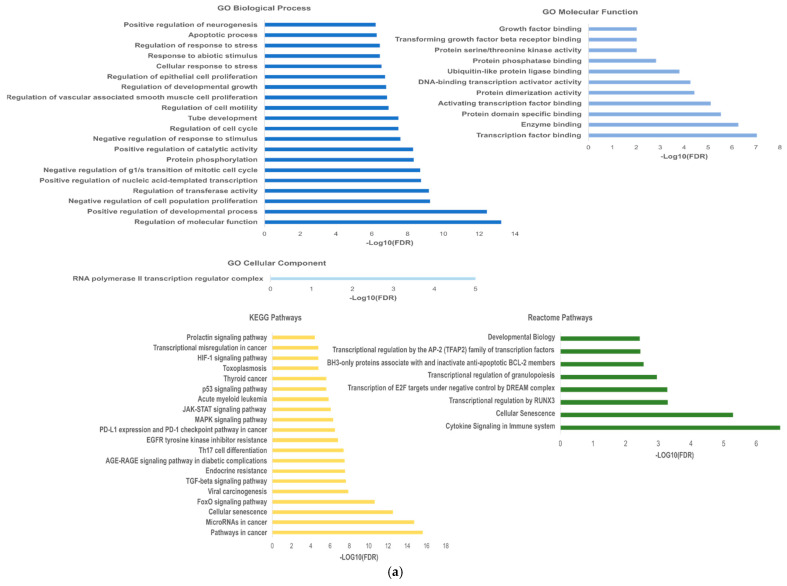

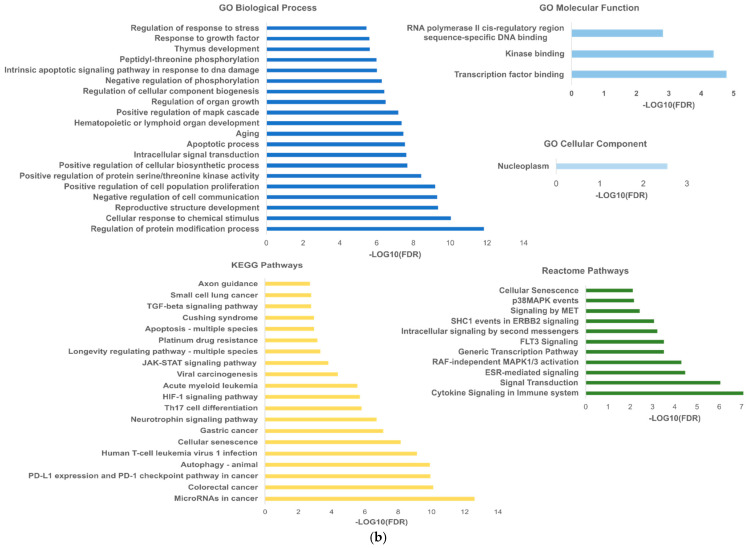
Enrichment analysis for hsa-miR-20a-5p targets (**a**) and hsa-miR-181a-5p targets (**b**).

For the functional enrichment analysis, we used a false discovery rate (FDR) threshold of *p* < 0.01, and the redundant terms were eliminated using a redundancy cutoff of 0.5, which resulted in a total of 236 enriched terms among the KEGG, Reactome and GO categories for hsa-miR-20a-5p and 218 enriched terms for hsa-miR-181a-5p. The enriched terms for each category after being filtered are represented in Figure 4, which shows only the top 20 enriched terms for the GO Biological Process and KEGG analysis.

Analyzing the functionally enriched terms for the hsa-miR-20a-5p targets, we could observe that the majority of the targets, including the CCND1, CCND2, SMAD7/4, E2F3/E2F1, STAT3, CDKN1A, MYC, PTEN and MAPK family, are involved in the regulation of central cell functions, such as regulation of cell proliferation, cell cycle and transcription, according to GO terms analysis, highlighting their involvement in hematopoietic or lymphoid organ development. Among the functionally enriched terms in the KEGG and Reactome pathways, we could find major cancer signaling pathways, such as FoxO, MAPK, JaK-STAT and p53, all of which are involved in NHL development. In the functional analysis of hsa-miR-181a-5p targets through GO terms analysis, we observed that several targets are involved in the cellular protein modification process, regulation of protein kinase activity, cell proliferation and apoptosis, with indications of their involvement in hematopoietic or lymphoid organ development. In the KEGG and Reactome pathways, we could find p53, JAK-STAT and MAPK signaling pathways and cytokine signaling in the immune system, which is highly associated with miRNA deregulation, demonstrating that all central signaling pathways are involved in the development and progression of NHL.

### 3.2. miRNA and lncRNA Expression Levels in NHL Patients’ Plasma Samples

**Figure 5:**
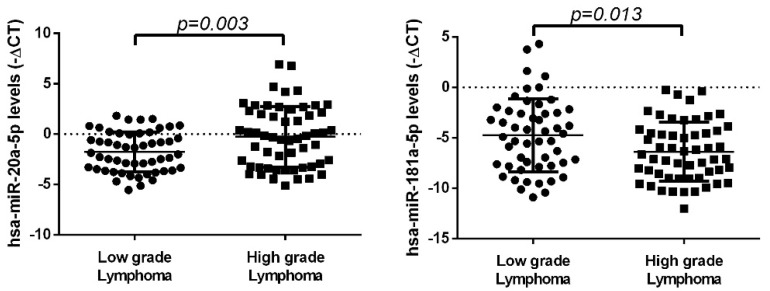
Expression levels of hsa-miR-20a-5p and hsa-miR-181a-5p in plasma samples of NHL patient groups according to lymphoma grade. Patients with high-grade lymphoma presented higher levels of hsa-miR-20a-5p and lower levels of hsa-miR-181a-5p compared to patients with low-grade lymphoma (*p* = 0.003 and 0.013, respectively). The graphics represent the −ΔCT of miRNA expression normalized to the endogenous control (mean ± SD).

We evaluated the plasma expression levels of hsa-miR-20a-5p and hsa-miR-181a-5p in 113 NHL patients using quantitative real-time PCR. The expression levels of hsa-miR-20a-5p were significantly higher (fold change = 2.83) in patients with high-grade lymphoma, whereas hsa-miR-181a-5p levels were significantly lower (fold change = 0.33) compared to low-grade lymphoma patients (*p* = 0.003 and 0.013, respectively) (Figure 5).

**Figure 6:**
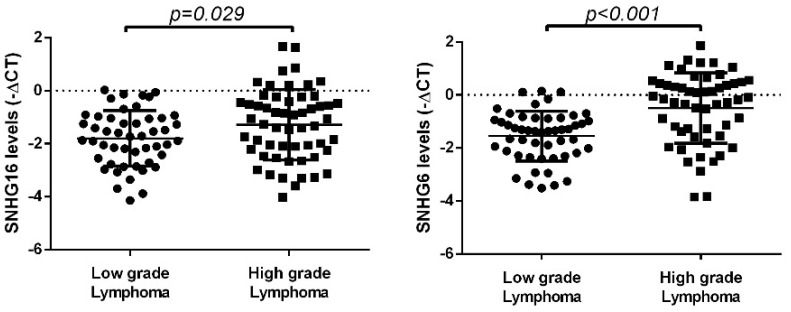
Expression levels of SNHG16 and SNHG6 in plasma samples of NHL patient groups according to lymphoma grade. SNHG16 and SNHG6 presented higher plasma levels in patients diagnosed with high-grade lymphoma compared to low-grade lymphoma (*p* = 0.029 and *p* < 0.001, respectively). The graphics represent the −ΔCT of miRNA expression normalized to the endogenous control (mean ± SD).

Next, the plasma expression levels of SNHG16 and SNHG6 were evaluated, and we observed higher plasma levels of both lncRNAs in patients with high-grade lymphomas compared to low-grade lymphomas (SNHG16: fold change = 1.63; SNHG6: fold change = 2.08) (*p* = 0.029 and *p* < 0.001, respectively) (Figure 6).

### 3.3. miRNAs and LncRNAs Plasma Levels According to IPI and FLIPI Score

**Figure 7:**
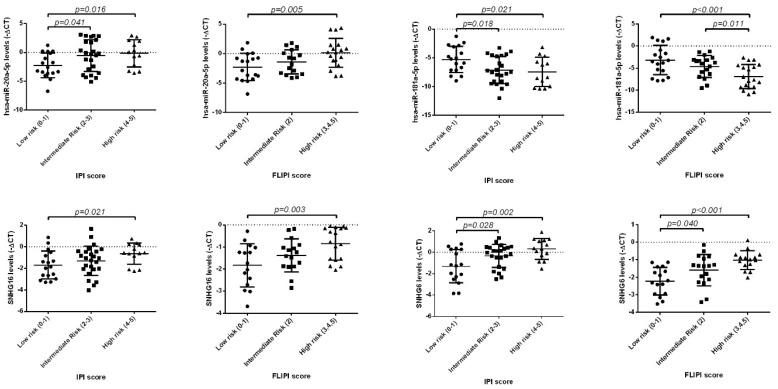
Expression levels of hsa-miR-20a-5p, hsa-miR-181a-5p, SNHG16 and SNHG6 in plasma samples of NHL patient groups according to IPI and FLIPI scores. High levels of hsa-miR-20a-5p, SNH16 and SNHG6 were associated with higher IPI and FLIPI scores, while low levels of hsa-miR-181a-5p were associated with higher IPI and FLIPI scores. The figures represent the −ΔCT of miRNA expression normalized to the endogenous control (mean ± SD).

We next examined the association of the plasma expression levels of hsa-miR-20a-5p, hsa-miR-181a-5p and lncRNAs SNHG16 and SNHG6 with the IPI scores and FLIPI scores of the NHL patients. The analysis revealed that higher levels of hsa-miR-20a-5p, SNHG16 and SNHG6 were associated with higher IPI and FLIPI scores. On the contrary, we observed a negative association between hsa-miR-181a-5p levels and IPI/FLIPI scores. Specifically, lower levels of hsa-miR-181a-5p were associated with higher IPI and FLIPI scores (Figure 7).

Given the ceRNAs expression analysis, three groups were established considering the combination of the plasma levels of each ceRNA pair, hsa-miR-20a-5p and SNHG16 plasma levels and hsa-miR-181a-5p and SNHG6 plasma levels, which allowed for the definition of high-, intermediate- and low-risk groups. For the analysis of the hsa-miR-20a-5p:SNHG16 pair, the low-risk group included patients with low levels of hsa-miR-20a-5p and SNHG16; the intermediate-risk group combined both patients with high hsa-miR-20a-5p and low SNHG16 levels and patients with low hsa-miR-20a-5p and high SNHG16 expression; and the high-risk group included patients with high levels of hsa-miR-20a-5p and high levels of SNHG16. Concerning the ceRNA pair hsa-miR-181a-5p:SNHG6, the low-risk group was composed of patients with high expression of hsa-miR-181a-5p and low expression of SNH6. The intermediate-risk group combined both patients with high hsa-miR-181a-5p and high SNHG6 expression and patients with low hsa-miR-181a-5p and low SNHG6 expression. The high-risk group combined patients with a low expression of hsa-miR-181a-5p and high expression of SNHG6 (Table 2).

### 3.4. miRNA and lncRNA Expression Levels in NHL Patients’ Plasma Samples according to Bone Marrow Involvement

Considering the risk groups previously defined based on the expression levels of each ceRNA pair, we could observe that high-risk profiles were associated with a higher risk of presenting BMI (Table 3). Patients with a low expression of hsa-miR-181a-5p with high expression levels of SNHG16 had a higher risk of having positive BMI (*p* = 0.010). Conversely, a higher risk of positive BMI was associated with high expression of hsa-miR-20a-5p and high expression of the SNHG6 profile (*p* = 0.023).

**Figure 8:**
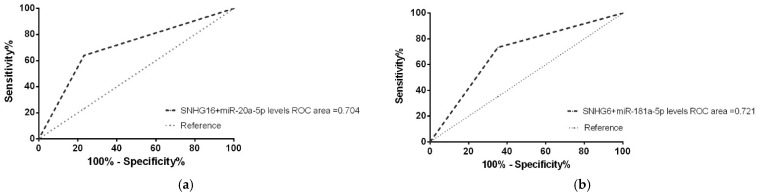
Plasma levels of ceRNA pair as biomarkers of BM involvement in NHL patients. ROC curve analysis of plasma levels of (**a**) SNHG16/hsa-miR-20a-5p and (**b**) SNHG6/hsa-miR-181a-5p ceRNA pair as diagnostic biomarkers differentiating positive BM involvement patients from negative BM involvement patients.

Next, ROC analysis was performed to explore the clinical value of each ceRNA pair levels in the diagnosis of BMI in NHL patients (Figure 8). Results show an AUC of 0.704 for SNHG16/hsa-miR-20a-5p (95% CI 0.579-0.829, *p* = 0.003) and, finally, an AUC of 0.721 for the ceRNA pair SNHG6/hsa-miR-181a-5p (95% CI 0.579-0.803, *p* = 0.002).

### 3.5. miRNAs’ and LncRNAs’ Impact on Overall Survival and Progression-Free Survival of NHL Patients

**Figure 9:**
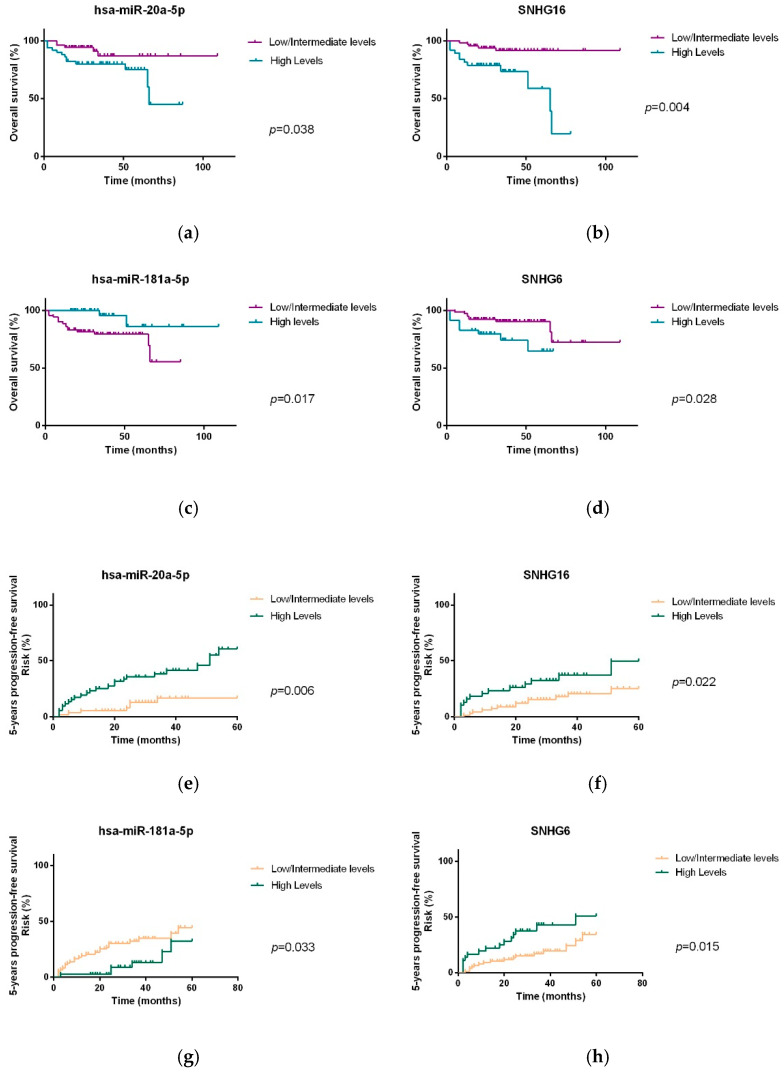
OS and PFS of NHL patients according to plasma levels of hsa-miR-20a-5p (**a**,**e**) hsa-miR-181a-5p (**c**,**g**), SNHG16 (**b**,**f**) and SNHG6 (**d**,**h**).

For the analysis of the OS and PFS, patients were divided in terciles according to the transcript levels using the –ΔCT values of each miRNA and lncRNA (high, intermediate and low levels), in order to analyze the association between the OS and PFS of NHL patients and the expression levels of hsa-miR-20a-5p, hsa-miR-181a-5p, SNHG16 and SNHG6. Kaplan–Meier analysis and log-rank tests revealed that lower plasma levels of hsa-miR-181a-5p were associated with lower OS and PFS (log-rank test: *p* = 0.017 and *p* = 0.033; HR: 0.200, *p* = 0.032; HR: 0.450, *p* = 0.048, respectively). On the other hand, higher plasma levels of hsa-miR-20a-5p, SHNG16 and SNHG6 were associated with worse OS and PFS (hsa-miR-20a-5p: *p* = 0.038 and *p* = 0.006, HR: 2.834 *p* = 0.037, HR: 3.898 *p* = 0.001; SNHG16: *p* = 0.004 and *p* = 0.022, HR: 4.481 *p* = 0.002, HR: 2.346 *p* = 0.029; SNHG6: *p* = 0.028 and *p* = 0.015, HR: 2.621 *p* = 0.043, HR: 2.325 *p* = 0.022, respectively) (Figure 9 and Table 4).

**Figure 10:**
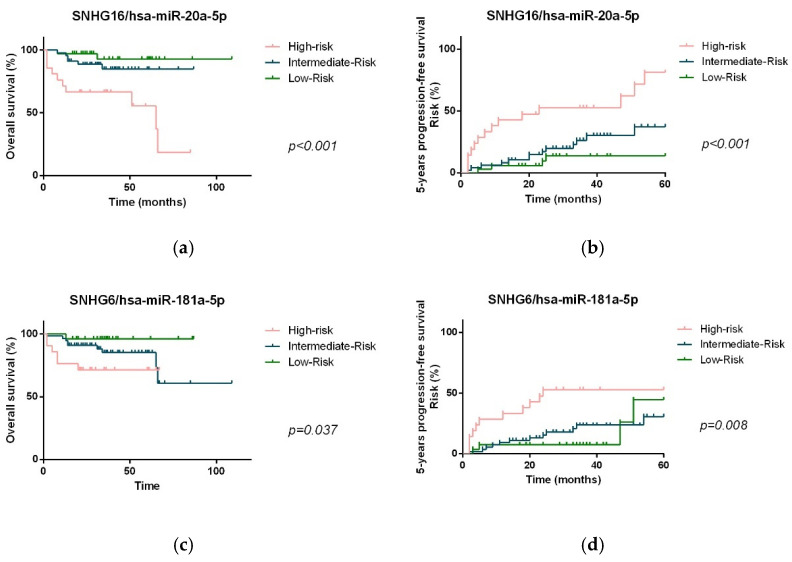
OS and PFS of NHL patients according to the defined risk groups based on the plasma levels of miRNA/lncRNA pairs, hsa-miR-20a-5p/SNHG16 (**a**,**b**) and hsa-miR-181a-5p/SNHG6 (**c**,**d**).

When considering the previously defined risk groups, we observed that the significance of the prognostic value was improved when expression levels of the miRNAs were combined with the respective lncRNA pair (Figure 10), showing that patients from the high-risk group from each ceRNA pair presented a significant lower OS and PFS than patients from the intermediate- and low-risk groups.

Multivariate analysis was conducted with significant clinical parameters associated with patients’ prognosis and revealed that high levels of both hsa-miR-20a-5p and SNHG16 were independent prognostic factors of poor patient prognosis regarding OS and PFS (OS: *p* = 0.034 and *p* = 0.007; PFS: *p* = 0.002 and *p* = 0.030) (Table 5 and Table 6). Moreover, low expression of hsa-miR-181a-5p and high expression of SNHG6 were independent prognostic factors associated with a poorer prognosis of NHL patients (OS: *p* = 0.035 and *p* = 0.047; PFS: *p* = 0.029 and *p* = 0.032) (Table 5 and Table 6). In order to compare the predictive ability of death and progression of the different variables, the c index was calculated for each model. Thus, Model 1, which incorporates age at diagnosis, tumor stage, tumor grade, presence of B symptoms, LDH serum levels and ECOG status, presented a predictive capacity with a c index of 0.608 for OS and 0.645 for PFS. Interestingly, when miRNA and corresponding lncRNA plasma levels were added to the previous variables, we observed an increase in the predictive capacity for each model created for both OS and PFS.

Therefore, an increased risk of death and disease progression was found in patients with low hsa-miR-181a-5p levels/high SNHG6 levels and high hsa-miR-20a-5p levels/high SNHG16 levels.

## 4. Discussion

Despite the remarkable improvements in outcomes, the intrinsic heterogeneity of NHL is reflected in the unpredictability of tumor behavior and, consequently, in the patient’s outcome [4,5,6]. Therefore, there is an urgent need to improve current patient prognosis stratification schemes by identifying molecular biomarkers that reflect tumor heterogeneity and clinical behavior. Given the precision medicine era that are currently in, it is now recognized that tissue biopsy, such as BM biopsy and BM aspirate, does not reliably reflect, either temporally and spatially, the whole genomic profile of the tumor. Moreover, they are considered invasive, complex procedures for obtaining samples, and are difficult to reproduce. Circulating tumor-associated components, such as miRNAs and lncRNAs, which can be easily assessed, have potential as lymphoma biomarkers, allowing for a personalized patient follow-up. Among the different lncRNA functions, there is an increasing interest in studying lncRNAs as miRNA sponges, which function as an extra layer of the post-transcriptional regulatory machinery to modulate miRNA-mediated gene expression. Moreover, integration of regulatory layers, such as the ceRNA network, represents not only an opportunity to dissect aberrant cellular functions behind the complex process of lymphomagenesis, but also an interesting approach to select more feasible biomarkers with functional relevance. The joint detection of lncRNAs and miRNAs can significantly improve the specificity and sensitivity of liquid-biopsy-based diagnosis and prognosis and increase our understanding of prognostic and predictive phenotypes, eventually leading to better patient follow-up. Therefore, in this study, we aimed to investigate for the first time the prognostic value of the ceRNA network by analyzing the lncRNA-miRNA dynamic pair’s expression in plasma samples of NHL patients, not only as predictors of the overall clinical outcomes, but also as biomarkers of important clinical determinants of disease progression, such as BMI. Our results show that hsa-miR-20a-5p, hsa-miR-181a-5p, SNHG16 and SNGH6 were differentially expressed in patients with high-grade lymphomas when compared to low-grade lymphoma patients, and their levels were statistically associated with IPI/FLIPI score, demonstrating their involvement in NHL prognosis. Moreover, the defined risk groups based on ceRNA pair levels could help determine the presence of BMI. Specifically, we determined whether NHL patients with high-risk expression profiles of hsa-miR-20a-5p/SNHG16 and hsa-miR-181a-5p/SNHG6 presented a higher risk of presenting a positive BMI. Moreover, we observed that high levels of hsa-miR-20a-5p, SNHG16 and SNHG6, and low levels of hsa-miR-181a-5p were associated with shorter OS and PFS, which was supported by the multivariate analysis that demonstrated that each transcript is an independent prognosis predictor. These results are further reinforced by the C-index analysis, where the models incorporating the ceRNA network expression analysis clearly improved the predictive capacity compared to the model which only considered the clinicopathological variables.

According to our bioinformatics analysis, lncRNA SNHG16 was identified as one of the hsa-miR-20a-5p sponges. SNHG16 has been widely described as an oncogenic factor in a variety of cancers, including B-cell lymphoma [34]. In fact, Zhu et al. reported that SNHG16 was upregulated in diffuse large B-cell lymphoma tissues and cell lines [34]. Functionally, SNHG16 was shown to induce cell proliferation, cell cycle and invasion and to inhibit apoptosis in the majority of human cancers [35,36,37]. However, the biological function of SNHG16 and its underlying mechanism in NHL are still unknown. SNHG16 transcription has been shown to be regulated by several transcription factors, such as c-Myc, STAT3 and TFAP2A [37,38]. Li et al. demonstrated that c-Myc recruits histone acetyltransferase and induces RNA polymerase II clearance to upregulate SNHG16 transcription, resulting in enhanced cell proliferation, migration and invasion and inhibited cell apoptosis in tumor cells [39]. Furthermore, in another study by Christensen et al., SNHG16 expression was positively modulated by Wnt-regulated transcription factors, including c-Myc [37]. On the other hand, SNHG16 can act as a scaffold on interchromatin clusters regulating gene expression, for example, by interacting with EZH2. The expression of p21 was shown to be directly inhibited by SNHG16 via recruitment of EZH2, which induces cell cycle, cell proliferation and inhibits apoptosis [36]. Moreover, silencing of SNHG16 leads to p21 upregulation and cyclin D1 and cyclin B1 downregulation [40]. SNHG16 can also post-transcriptionally regulate gene expression by acting as a ceRNA sequestering miRNAs. In diffuse large B-cell lymphoma, SNHG16 was shown to sponge miR-497-5p, releasing PIM1 from miR-497-5p-mediated inhibition, which promotes proliferation, cell cycle and inhibits apoptosis of lymphoma cells [34]. Knockdown of SNHG16 in multiple myeloma cells suppressed cell proliferation, induced cell arrest and promoted the apoptosis via inducing cleaved-Caspase-3, cleaved-Caspase-9, Foxa3a and Bax expression, while inhibiting CCND1, Bcl-2, Cyclin D1, PI3K and p-AKT. The SNHG16 effect was shown to be due to sponging miR-342-3p [41]. Li et al. reported that SNHG16 competitively binds to miR-4500, upregulating STAT3 and leading to cell proliferation, migration, invasion and the epithelial–mesenchymal transition process as well as inhibiting cell apoptosis [42]. Zhang et al. reported that SNHG16 acts as a ceRNA, sponging miR-17-5p to upregulate p62, which culminates in the activation of the mTOR/PI3K/AKt pathway and NF-κB signaling to promote proliferation of tumor cells and to repress apoptosis [43]. Recently, it was shown that SNHG16 contains a binding site for hsa-miR-20a-5p, acting as a ceRNA to inhibit its inhibitory function.

MiR-20a-5p is a well-established NHL-associated miRNA belonging to the miR-17-92 cluster, which regulates different stages of B-cell development and central tolerance [22,23,24]. According to our results, similarly to its target lncRNA SNHG16, hsa-miR-20a-5p was also found to be upregulated in the plasma samples of NHL patients. In fact, the miR-17-92 cluster is frequently found to be overexpressed due to genomic amplification (q31.3) in several lymphomas, including DLBCL and FL [44]. Moreover, overexpression of miR-20a is associated with c-Myc expression, whose concomitant expression promoted the onset of tumors and increased their growth in a mouse model of B cell lymphoma [45]. c-Myc is not only involved in the transcription of SNHG16, but can also bind to the promoter region of miR-20a, inducing its expression [46]. Diverse targets of has-miR-20a-5p have been identified that could explain its oncogenic role in NHL. Most notably, miR-20a targets PTEN, whose inhibition results in the activation of the PI3K/AKT pathway, one of the central pathways in NHL development [47,48,49]. Additionally, miR-20a targets CDKN1A/p21, which is a cell cycle inhibitor enforcing cell cycle arrest in G1/S [50]. The oncogene function of miR-20a-5p was also shown to be associated with the inhibition of early growth response (EGR)2, thus promoting cell proliferation and cell cycle progression [51]. Therefore, we propose that the upregulation of both hsa-miR-20a-5p and the corresponding lncRNA pair SNHG16 is due to the preponderant action of the deregulated MYC in lymphomas, which acts as a transcription factor of both genes in order to promote lymphoma cells’ proliferation and survival.

Concerning hsa-miR-181a-5p, SNHG6 was found to be one of the lncRNAs acting as a ceRNA. SNHG6 has been found to be significantly overexpressed in different tumors, and is also associated with poor clinical outcomes [52]. In a recent study analyzing lncRNA-mediated ceRNA networks in Hodgkin lymphoma, SNHG6 was reported as upregulated, and highly associated with patients’ relapse [53]. Therefore, SNHG6 has been characterized as an oncogenic lncRNA involved in the regulation of cell differentiation, proliferation, apoptosis and multidrug resistance [54,55]. Similar to SNHG16, SNHG6 regulates gene expression transcriptionally by recruiting EZH2 to promoter regions of different tumor suppressor genes, such as P27 and P21, and represses their expression through methylation of their promoters [55,56]. Moreover, SNHG6 was identified as a molecular sponge of miR-101, miR-214 and miR-4465, all of which target EZH2 [57,58,59]. Therefore, SNHG6 can modulate the function of EZH2 at multiple levels. By competitively sponging miR-101, SNHG6 also regulates the expression of ZEB1, promoting cell migration and EMT [55]. As determined by our bioinformatics analysis, SNHG6 was identified as targeting miR-181a. In fact, SNHG6 was also shown to act as a molecular decoy for all four members of the miR-181 family. Overexpression of SNHG6 represses miR-181a, which in turn induces JAK2 expression and promotes tumor cell proliferation [60]. Moreover, SNHG6-mediated inhibition of miR-181a was shown to induce proliferation, cell cycle progression and migration and invasion and inhibits apoptosis via upregulation of E2F5 [61]. Using a bioinformatics analysis, we identified an miR-181a-related PPI network linked to proteins such as E2F5, CDKN1B, the MAPK family, BCL-2/BCL2L11 and MET signaling (KRAS and STAT3). This PPI network was enriched in cytokine signaling in the immune system, positive regulation of cell proliferation, cell cycle and deregulation of miRNAs in cancer pathogenesis, as demonstrated in the KEGG and Reactome pathway analysis. Interestingly, when analyzing the Go terms, one of the most enriched was hematopoietic or lymphoid organ development, emphasizing the involvement of miR-181a in the hematologic system and its possible involvement in NHL pathogenesis. In fact, a study by Kozloski et al. reported that miR-181a is a negative regulator of the NF-kB signaling pathway in DLBCL cells, inhibiting tumor cell proliferation and viability [62]. Overall, SNHG6 and miR-181a were shown to directly interact with each, in which SNHG6 functions as an miR-181a decoy; furthermore, there was an overlap of downstream targets, specially involving the MAPK signaling pathway, whose aberrant activation has been previously described in NHL [63]. Therefore, we hypothesize a potential involvement of SNHG6-mediated suppression of miR-181a leading to promotion of proliferation signaling networks and inhibition of apoptosis assisting in NHL progression.

## 5. Conclusions

Although several studies have identified miRNAs and lncRNAs in NHL, the number of functional studies on miRNA-lncRNA interplay is still limited, which precludes the defined diagnostic and prognostic importance of the ceRNA network in NHL patients. This approach narrows the scope of research and enhances the prediction accuracy to identify candidate biomarkers with great potential for the diagnosis, prognosis and as therapeutic targets for NHL patients. Moreover, the majority of studies focus on the expression profile in tissue samples and cell lines, with very few studies analyzing their expression levels in the circulation of NHL patients and viewing them as novel less invasive complementary biomarkers in NHL prognosis. Plasma levels of the miR-20a/SNHG16 pair and miR-181a/SNHG6 could serve as new prognostic biomarkers for NHL. In our study, plasma levels of hsa-miR-20a-5/SNHG16 and hsa-miR-181a-5p/SNHG6 were associated with patient clinical outcome, where patients with high hsa-miR-20a-5p/high SNHG16 and low hsa-miR-181a-5p/high SNHG6 presented worse OS and PFS. Therefore, our results indicate that these ceRNA pairs could function as NHL prognostic biomarkers to better identify risk patients and consequently could help improve patients’ management. Despite the new approach of our study to identify new circulating NHL biomarkers by introducing the analysis of ceRNA network components, it would be interesting to also analyze the expression of the target mRNAs involved in the identified ceRNA networks and, consequently, analyze their clinical value. Additionally, future studies should focus on validating these results on a larger patient cohort and clarify the biological function of the identified transcripts by performing in vitro studies that permit the modulation of the transcripts’ expression and, consequently, investigate their influence on tumor cells’ properties.

## Figures and Tables

**Table 1:** Patients’ clinicopathologic characteristics.

Clinical–Pathological Characteristics	N (%) N = 113
**Age**	
≤60 years	53 (46.9%)
>60 years	60 (53.1%)
**Gender**	
Female	57 (50.4%)
Male	56 (49.6%)
**Grade**	
Low (indolent)	55 (48.7%)
High (aggressive)	58 (51.3%)
**Subtype of NHL**	
Follicular	40 (35.4%)
Diffuse large B cell	58 (51.3%)
Marginal Zone	15 (13.3%)
**Stage**	
I/II	43 (38.1%)
III/IV	70 (61.9%)
**LDH serum levels**	
Normal	67 (59.3%)
High	45 (39.8%)
Unknown	1 (0.9%)
**ECOG**	
0–1	97 (85.8%)
≥2	14 (12.4%)
**Unknown**	2 (1.8%)
B symptoms	
Absent	80 (70.8%)
Present	33 (29.2%)
**IPI Score (high-grade tumors)**	
Low risk (0–1)	17 (29.3%)
Intermediate risk (2–3)	25 (43.1%)
High risk (4–5)	14 (24.1%)
Unknown	2 (3.4%)
**FLIPI score (low-grade tumors)**	
Low risk (0–1)	18 (32.7%)
Intermediate risk (2)	18 (32.7%)
High risk (3, 4, 5)	19 (34.5%)
**BM involvement**	
Negative	81 (71.7%)
Positive	32 (28.3%)

**Table 2:** Definition of high-, intermediate- and low-risk groups considering the combination of the plasma levels of each ceRNA pair, hsa-miR-20a-5p/SNHG16 and hsa-miR-181a-5p/SNHG6 plasma levels(upwards arrow represents transcript upregulation, downwards arrow represents transcript downregulation).

Groups	hsa-miR-20a-5p/SNHG16	hsa-miR-181a-5p/SNHG6
Low risk	↓hsa-miR-20a-5p + ↓SNHG16	↑hsa-miR-181a-5p + ↓SNHG6
Intermediate risk	↓hsa-miR-20a-5p + ↓SNHG16 ↓hsa-miR-20a-5p + ↑SNHG16	↑hsa-miR-181a-5p + ↑SNHG6 ↓hsa-miR-181a-5p + ↓SNHG6
High risk	↑hsa-miR-20a-5p + ↑SNHG16	↓hsa-miR-181a-5p + ↑SNHG6

**Table 3:** The association between the risk groups considering the plasma levels of each ceRNA pair and the presence of bone marrow involvement in NHL patients.

Risk Groups	n	Negative BMI (n)	Positive BMI (n)	OR (95% CI)	*p*
hsa-miR-20a-5p/SNHG16					0.023
Low/intermediate risk	59	48	11	1	
High risk	36	21	14	2.91 (1.14–7.46)	
hsa-miR-181a-5p/SNHG6					0.010
Low/intermediate risk	56	46	28	1	
High risk	48	10	20	3.28 (1.35–8.02)	

**Table 4:** The results of the univariate analysis for overall survival and 5-year progression-free survival.

Characteristic	OS		5-Year PFS	
	HR (95% CI)	*p*	HR (95% CI)	*p*
miR-20a levels (Low/Inter vs. High)	2.834 (1.007–7.979)	0.037	3.898 (1.676–9.045)	0.001
miR-181a levels (Low/Inter vs. High)	0.200 (0.046–0.871)	0.032	0.450 (0.194–1.046)	0.048
SNHG16 levels (Low/Inter vs. High)	4.481 (1.672–12.005)	0.002	2.346 (1.100–5.004)	0.029
SNHG6 levels (Low/Inter vs. High)	2.621 (1.029–6.675)	0.043	2.325 (1.147–4.710)	0.022

**Table 5:** The results of the multivariate analysis for overall survival regarding each ceRNA network pair.

Characteristic	OS		
	HR (95% CI)	*p*	c Index
**Model 1**			
Age (≤60 years vs. >60 years)	2.446 (0.788–7.596)	0.122	0.608
Lymphoma grade (Low vs. High)	2.338 (1.112–4.915)	0.025	
B symptoms (Absent vs. Present)	1.116 (0.355–3.508)	0.081	
Stage (I/II vs. III/IV)	3.311 (1.705–5.546)	0.029	
ECOG (0–1 vs. ≥2)	2.418 (1.326–5.722)	0.036	
LDH levels (normal vs. high)	2.696(1.656–4.388)	0.037	
**Model 2**			
Age (≤60 years vs. >60 years)	2.584 (0.826–19.353)	0.105	0.689
Lymphoma grade (Low vs. High)	3.824 (1.167–12.536)	0.027	
B symptoms (Absent vs. Present)	1.743 (0.197–2.805)	0.661	
Stage (I/II vs. III/IV)	3.999 (0.826–9.353)	0.085	
ECOG (0–1 vs. ≥2)	4.447 (1.221–6.200)	0.024	
LDH levels (normal vs. high)	1.887 (0.676–5.267)	0.225	
hsa-miR-20a-5p levels (Low/Inter vs. High)	3.129 (1.090–8.984)	0.034	
SNHG16 levels (Low/Inter vs. High)	4.393 (1.488–8.969)	0.007	
**Model 3**			
Age (≤60 years vs. >60 years)	2.589 (0.790–8.426)	0.116	0.703
Lymphoma grade (Low vs. High)	3.717 (1.097–12.590)	0.035	
B symptoms (Absent vs. Present)	1.744 (0.210–2.638)	0.647	
Stage (I/II vs. III/IV)	2.489 (1.080–5.486)	0.040	
ECOG (0–1 vs. ≥2)	4.701 (1.349–6.381)	0.015	
LDH levels (normal vs. high)	1.043 (0.321–3.387)	0.244	
hsa-miR-181a-5p levels (Low/Inter vs. High)	0.207(0.41–1.032)	0.035	
SNHG6 levels (Low/Inter vs. High)	2.801 (1.015–7.728)	0.047	

**Table 6:** The results of the multivariate analysis for 5-year progression-free survival for each ceRNA network pair.

Characteristic	5-Year PFS		
	HR (95% CI)	*p*	c Index
**Model 1**			
Age (≤60 years vs. >60 years)	2.025 (1.040–3.944)	0.038	0.645
Lymphoma grade (Low vs. High)	2.315 (1.403– 3.820)	0.001	
B symptoms (Absent vs. Present	1.644 (0.312–1.329)	0.234	
Stage (I/II vs. III/IV)	2.437 (1.149–5.167)	0.020	
ECOG (0–1 vs. ≥2)	1.402 (1.040–7.720)	0.057	
LDH levels (normal vs. high)	1.509 (0.855–2.662)	0.156	
**Model 2**			
Age (≤60 years vs. >60 years)	2.456 (0.993–6.073)	0.052	0.810
Lymphoma grade (Low vs. High)	2.223 (1.021–4.840)	0.044	
B symptoms (Absent vs. Present)	1.762 (0.256–2.263)	0.624	
Stage (I/II vs. III/IV)	3.994 (1.378–11.580)	0.011	
ECOG (0–1 vs. ≥2)	3.205 (1.151–8.921)	0.026	
LDH levels (normal vs. high)	2.158 (0.989–4.714)	0.053	
hsa-miR-20a-5p levels (Low/Inter vs. High)	3.875 (1.619–9.279)	0.002	
SNHG16 levels (Low/Inter vs. High)	3.658 (1.410–9.491)	0.030	
**Model 3**			
Age (≤60 years vs. >60 years)	2.760 (1.127–6.760)	0.026	0.709
Lymphoma grade (Low vs. High)	2.368 (1.114–5.035)	0.025	
B symptoms (Absent vs. Present)	1.968 (0.935–4.142)	0.074	
Stage (I/II vs. III/IV)	3.052 (1.027–9.065)	0.045	
ECOG (0–1 vs. ≥2)	2.901 (2.939–4.216)	0.021	
LDH levels (normal vs. high)	1.433 (0.668–3.075)	0.355	
hsa-miR-181a-5p levels (Low/Inter vs. High)	0.374 (0.154–0.906)	0.029	
SNHG6 levels (Low/Inter vs. High)	2.183 (0.142–0.783)	0.032	

## Data Availability

No application.

## References

[1] Shaffer A.L., Young R.M., Staudt L.M. (2012). Pathogenesis of Human B Cell Lymphomas. Annu. Rev. Immunol..

[2] Bray F., Ferlay J., Soerjomataram I., Siegel R.L., Torre L.A., Jemal A. (2018). Global cancer statistics 2018: GLOBOCAN estimates of incidence and mortality worldwide for 36 cancers in 185 countries. CA A Cancer J. Clin..

[3] Armitage J.O., Gascoyne R.D., Lunning M.A., Cavalli F. (2017). Non-Hodgkin lymphoma. Lancet.

[4] Klener P., Klanova M. (2020). Drug Resistance in Non-Hodgkin Lymphomas. Int. J. Mol. Sci..

[5] Rovira J., Valera A., Colomo L., Setoain X., Rodríguez S., Martínez-Trillos A., Giné E., Dlouhy I., Magnano L., Gaya A. (2015). Prognosis of patients with diffuse large B cell lymphoma not reaching complete response or relapsing after frontline chemotherapy or immunochemotherapy. Ann. Hematol..

[6] Crump M., Neelapu S.S., Farooq U., Van Den Neste E., Kuruvilla J., Westin J., Link B.K., Hay A., Cerhan J.R., Zhu L. (2017). Outcomes in refractory diffuse large B-cell lymphoma: Results from the international SCHOLAR-1 study. Blood.

[7] (1993). A predictive model for aggressive non-Hodgkin’s lymphoma. N. Engl. J. Med..

[8] Solal-Céligny P., Roy P., Colombat P., White J., Armitage J.O., Arranz-Saez R., Au W.Y., Bellei M., Brice P., Caballero D. (2004). Follicular lymphoma international prognostic index. Blood.

[9] Ikeda S., Tsunoda S., Koyama D., Suzuki M., Sukegawa M., Misawa K., Hojo H., Zhu X., Utano K., Ohta M. (2021). Femoral marrow MRI is a non-invasive, non-irradiated and useful tool for detecting bone marrow involvement in non-Hodgkin lymphoma. J. Clin. Exp. Hematop. JCEH.

[10] Ghafouri-Fard S., Esmaeili M., Taheri M. (2020). Expression of non-coding RNAs in hematological malignancies. Eur. J. Pharmacol..

[11] Sole C., Arnaiz E., Manterola L., Otaegui D., Lawrie C.H. (2019). The circulating transcriptome as a source of cancer liquid biopsy biomarkers. Semin. Cancer Biol..

[12] Bartel D.P. (2004). MicroRNAs: Genomics, biogenesis, mechanism, and function. Cell.

[13] O’Brien J., Hayder H., Zayed Y., Peng C. (2018). Overview of MicroRNA Biogenesis, Mechanisms of Actions, and Circulation. Front. Endocrinol..

[14] Solé C., Arnaiz E., Lawrie C.H. (2018). MicroRNAs as Biomarkers of B-cell Lymphoma. Biomarker Insights.

[15] Kopp F., Mendell J.T. (2018). Functional Classification and Experimental Dissection of Long Noncoding RNAs. Cell.

[16] Karstensen K.T., Schein A., Petri A., Bøgsted M., Dybkær K., Uchida S., Kauppinen S. (2021). Long Non-Coding RNAs in Diffuse Large B-Cell Lymphoma. Non-Coding RNA.

[17] Statello L., Guo C.-J., Chen L.-L., Huarte M. (2021). Gene regulation by long non-coding RNAs and its biological functions. Nat. Rev. Mol. Cell Biol..

[18] Salmena L., Poliseno L., Tay Y., Kats L., Pandolfi P.P. (2011). A ceRNA hypothesis: The Rosetta Stone of a hidden RNA language?. Cell.

[19] Li Q., Li B., Lu C.-L., Wang J.-Y., Gao M., Gao W. (2020). LncRNA LINC01857 promotes cell growth and diminishes apoptosis via PI3K/mTOR pathway and EMT process by regulating miR-141-3p/MAP4K4 axis in diffuse large B-cell lymphoma. Cancer Gene Therapy.

[20] Baytak E., Gong Q., Akman B., Yuan H., Chan W.C., Kucuk C. (2017). Whole transcriptome analysis reveals dysregulated oncogenic lncRNAs in natural killer/T-cell lymphoma and establishes MIR155HG as a target of PRDM1. Tumour Biol. J. Int. Soc. Oncodevelopmental Biol. Med..

[21] Xiao Y., Jiao C., Lin Y., Chen M., Zhang J., Wang J., Zhang Z. (2017). lncRNA UCA1 Contributes to Imatinib Resistance by Acting as a ceRNA Against miR-16 in Chronic Myeloid Leukemia Cells. DNA Cell Biol..

[22] Neat M.J., Foot N., Jenner M., Goff L., Ashcroft K., Burford D., Dunham A., Norton A., Lister T.A., Fitzgibbon J. (2001). Localisation of a novel region of recurrent amplification in follicular lymphoma to an∼ 6.8 Mb region of 13q32-33. Genes Chromosomes Cancer.

[23] Monni O., Oinonen R., Elonen E., Franssila K., Teerenhovi L., Joensuu H., Knuutila S. (1998). Gain of 3q and deletion of 11q22 are frequent aberrations in mantle cell lymphoma. Genes Chromosomes Cancer.

[24] Rao P.H., Houldsworth J., Dyomina K., Parsa N.Z., Cigudosa J.C., Louie D.C., Popplewell L., Offit K., Jhanwar S.C., Chaganti R.S.K. (1998). Chromosomal and Gene Amplification in Diffuse Large B-Cell Lymphoma. Blood.

[25] Jin H.Y., Oda H., Lai M., Skalsky R.L., Bethel K., Shepherd J., Kang S.G., Liu W.-H., Sabouri-Ghomi M., Cullen B.R. (2013). MicroRNA-17∼92 plays a causative role in lymphomagenesis by coordinating multiple oncogenic pathways. EMBO J..

[26] Chen C.-Z., Li L., Lodish H.F., Bartel D.P. (2004). MicroRNAs modulate hematopoietic lineage differentiation. science.

[27] Xiao C., Calado D.P., Galler G., Thai T.-H., Patterson H.C., Wang J., Rajewsky N., Bender T.P., Rajewsky K. (2007). MiR-150 Controls B Cell Differentiation by Targeting the Transcription Factor c-Myb. Cell.

[28] Okuyama K., Ikawa T., Gentner B., Hozumi K., Harnprasopwat R., Lu J., Yamashita R., Ha D., Toyoshima T., Chanda B. (2013). MicroRNA-126–mediated control of cell fate in B-cell myeloid progenitors as a potential alternative to transcriptional factors. Proc. Natl. Acad. Sci. USA.

[29] Li J.H., Liu S., Zhou H., Qu L.H., Yang J.H. (2014). starBase v2.0: Decoding miRNA-ceRNA, miRNA-ncRNA and protein-RNA interaction networks from large-scale CLIP-Seq data. Nucleic Acids Res..

[30] Huang H.Y., Lin Y.C., Li J., Huang K.Y., Shrestha S., Hong H.C., Tang Y., Chen Y.G., Jin C.N., Yu Y. (2020). miRTarBase 2020: Updates to the experimentally validated microRNA-target interaction database. Nucleic Acids Res..

[31] Shannon P., Markiel A., Ozier O., Baliga N.S., Wang J.T., Ramage D., Amin N., Schwikowski B., Ideker T. (2003). Cytoscape: A software environment for integrated models of biomolecular interaction networks. Genome Res..

[32] Fernandes M., Marques H., Teixeira A.L., Medeiros R. (2022). ceRNA Network of lncRNA/miRNA as Circulating Prognostic Biomarkers in Non-Hodgkin Lymphomas: Bioinformatic Analysis and Assessment of Their Prognostic Value in an NHL Cohort. Int. J. Mol. Sci..

[33] Zimta A.-A., Tigu A.B., Braicu C., Stefan C., Ionescu C., Berindan-Neagoe I. (2020). An Emerging Class of Long Non-coding RNA With Oncogenic Role Arises From the snoRNA Host Genes. Front. Oncol..

[34] Zhu Q., Li Y., Guo Y., Hu L., Xiao Z., Liu X., Wang J., Xu Q., Tong X. (2019). Long non-coding RNA SNHG16 promotes proliferation and inhibits apoptosis of diffuse large B-cell lymphoma cells by targeting miR-497-5p/PIM1 axis. J. Cell Mol. Med..

[35] Zhao W., Fu H., Zhang S., Sun S., Liu Y. (2018). LncRNA SNHG16 drives proliferation, migration, and invasion of hemangioma endothelial cell through modulation of miR-520d-3p/STAT3 axis. Cancer Med..

[36] Cao X., Xu J., Yue D. (2018). LncRNA-SNHG16 predicts poor prognosis and promotes tumor proliferation through epigenetically silencing p21 in bladder cancer. Cancer Gene Ther..

[37] Christensen L.L., True K., Hamilton M.P., Nielsen M.M., Damas N.D., Damgaard C.K., Ongen H., Dermitzakis E., Bramsen J.B., Pedersen J.S. (2016). SNHG16 is regulated by the Wnt pathway in colorectal cancer and affects genes involved in lipid metabolism. Mol. Oncol..

[38] Zhang G., Ma A., Jin Y., Pan G., Wang C. (2019). LncRNA SNHG16 induced by TFAP2A modulates glycolysis and proliferation of endometrial carcinoma through miR-490-3p/HK2 axis. Am. J. Transl. Res..

[39] Li S., Zhang S., Chen J. (2019). c-Myc induced upregulation of long non-coding RNA SNHG16 enhances progression and carcinogenesis in oral squamous cell carcinoma. Cancer Gene Ther..

[40] Zhou X.Y., Liu H., Ding Z.B., Xi H.P., Wang G.W. (2020). lncRNA SNHG16 Exerts Oncogenic Functions in Promoting Proliferation of Glioma Through Suppressing p21. Pathol. Oncol. Res. POR.

[41] Yang X., Huang H., Wang X., Liu H., Liu H., Lin Z. (2020). Knockdown of lncRNA SNHG16 suppresses multiple myeloma cell proliferation by sponging miR-342-3p. Cancer Cell Int..

[42] Lin Q., Zheng H., Xu J., Zhang F., Pan H. (2019). LncRNA SNHG16 aggravates tumorigenesis and development of hepatocellular carcinoma by sponging miR-4500 and targeting STAT3. J. Cell Biochem..

[43] Zhong J.H., Xiang X., Wang Y.Y., Liu X., Qi L.N., Luo C.P., Wei W.E., You X.M., Ma L., Xiang B.D. (2020). The lncRNA SNHG16 affects prognosis in hepatocellular carcinoma by regulating p62 expression. J. Cell. Physiol..

[44] Ota A., Tagawa H., Karnan S., Tsuzuki S., Karpas A., Kira S., Yoshida Y., Seto M. (2004). Identification and characterization of a novel gene, C13orf25, as a target for 13q31-q32 amplification in malignant lymphoma. Cancer Res..

[45] He L., Thomson J.M., Hemann M.T., Hernando-Monge E., Mu D., Goodson S., Powers S., Cordon-Cardo C., Lowe S.W., Hannon G.J. (2005). A microRNA polycistron as a potential human oncogene. Nature.

[46] Mu P., Han Y.C., Betel D., Yao E., Squatrito M., Ogrodowski P., de Stanchina E., D’Andrea A., Sander C., Ventura A. (2009). Genetic dissection of the miR-17~92 cluster of microRNAs in Myc-induced B-cell lymphomas. Genes Dev..

[47] Gao X., Qin T., Mao J., Zhang J., Fan S., Lu Y., Sun Z., Zhang Q., Song B., Li L. (2019). PTENP1/miR-20a/PTEN axis contributes to breast cancer progression by regulating PTEN via PI3K/AKT pathway. J. Exp. Clin. Cancer Res..

[48] Rao E., Jiang C., Ji M., Huang X., Iqbal J., Lenz G., Wright G., Staudt L.M., Zhao Y., McKeithan T.W. (2012). The miRNA-17∼92 cluster mediates chemoresistance and enhances tumor growth in mantle cell lymphoma via PI3K/AKT pathway activation. Leukemia.

[49] Jiang Y., Chang H., Chen G. (2018). Effects of microRNA-20a on the proliferation, migration and apoptosis of multiple myeloma via the PTEN/PI3K/AKT signaling pathway. Oncol. Lett..

[50] Zhou B.B., Elledge S.J. (2000). The DNA damage response: Putting checkpoints in perspective. Nature.

[51] Zhuo W., Ge W., Meng G., Jia S., Zhou X., Liu J. (2015). MicroRNA-20a promotes the proliferation and cell cycle of human osteosarcoma cells by suppressing early growth response 2 expression. Mol. Med. Rep..

[52] Zhao S., Zhu H., Jiao R., Wu X., Ji G., Zhang X. (2020). Prognostic and clinicopathological significance of SNHG6 in human cancers: A meta-analysis. BMC Cancer.

[53] Liang Y., Zhu H., Chen J., Lin W., Li B., Guo Y. (2020). Construction of relapse-related lncRNA-mediated ceRNA networks in Hodgkin lymphoma. Arch. Med. Sci..

[54] Fan R.H., Guo J.N., Yan W., Huang M.D., Zhu C.L., Yin Y.M., Chen X.F. (2018). Small nucleolar host gene 6 promotes esophageal squamous cell carcinoma cell proliferation and inhibits cell apoptosis. Oncol. Lett..

[55] Yan K., Tian J., Shi W., Xia H., Zhu Y. (2017). LncRNA SNHG6 is Associated with Poor Prognosis of Gastric Cancer and Promotes Cell Proliferation and EMT through Epigenetically Silencing p27 and Sponging miR-101-3p. Cell. Physiol. Biochem..

[56] Li Z., Qiu R., Qiu X., Tian T. (2018). SNHG6 Promotes Tumor Growth via Repression of P21 in Colorectal Cancer. Cell. Physiol. Biochem..

[57] Wang J., Yang X., Li R., Zhang R., Hu D., Zhang Y., Gao L. (2020). LncRNA SNHG6 Inhibits Apoptosis by Regulating EZH2 Expression via the Sponging of MiR-101-3p in Esophageal Squamous-Cell Carcinoma. OncoTargets Ther..

[58] Wu Y., Deng Y., Guo Q., Zhu J., Cao L., Guo X., Xu F., Weng W., Ju X., Wu X. (2019). Long non-coding RNA SNHG6 promotes cell proliferation and migration through sponging miR-4465 in ovarian clear cell carcinoma. J. Cell Mol. Med..

[59] Xu M., Chen X., Lin K., Zeng K., Liu X., Xu X., Pan B., Xu T., Sun L., He B. (2019). lncRNA SNHG6 regulates EZH2 expression by sponging miR-26a/b and miR-214 in colorectal cancer. J. Hematol. Oncol..

[60] Lai F., Deng W., Fu C., Wu P., Cao M., Tan S. (2020). Long non-coding RNA SNHG6 increases JAK2 expression by targeting the miR-181 family to promote colorectal cancer cell proliferation. J. Gene Med..

[61] Yu C., Sun J., Leng X., Yang J. (2019). Long noncoding RNA SNHG6 functions as a competing endogenous RNA by sponging miR-181a-5p to regulate E2F5 expression in colorectal cancer. Cancer Manag. Res..

[62] Kozloski G.A., Jiang X., Bhatt S., Ruiz J., Vega F., Shaknovich R., Melnick A., Lossos I.S. (2016). miR-181a negatively regulates NF-κB signaling and affects activated B-cell–like diffuse large B-cell lymphoma pathogenesis. Blood.

[63] Islam S., Qi W., Morales C., Cooke L., Spier C., Weterings E., Mahadevan D. (2017). Disruption of Aneuploidy and Senescence Induced by Aurora Inhibition Promotes Intrinsic Apoptosis in Double Hit or Double Expressor Diffuse Large B-cell Lymphomas. Mol. Cancer Ther..

